# Predictors of Change in Employment Status and Associations with Quality of Life: A Prospective International Study of People with Multiple Sclerosis

**DOI:** 10.1007/s10926-019-09850-5

**Published:** 2019-08-07

**Authors:** Claudia H. Marck, Zoe Aitken, Steve Simpson, Tracey J. Weiland, Anne Kavanagh, George A. Jelinek

**Affiliations:** 1 Disability and Health Unit, The Melbourne School of Population and Global Health, The University of Melbourne, Parkville, Australia; 2 Neuroepidemiology Unit, The Melbourne School of Population and Global Health, The University of Melbourne, Parkville, Australia; 3 Menzies Institute for Medical Research, University of Tasmania, Hobart, Australia

**Keywords:** Multiple sclerosis, Employment, Prospective, Quality of life, Job loss

## Abstract

*Purpose* This prospective international study aimed to assess the changes in employment, and predictors thereof, and associated change in mental health quality of life in people with multiple sclerosis (MS). *Methods* People with MS were recruited online through social media, forums and newsletters to complete an online English-language survey in 2012 and again in 2015, to assess changes in employment and clinical characteristics. *Results* 1276 people with MS of working age were included of whom 35.9% were employed full time, 25.6% part-time, 3.1% were unemployed and seeking employment, 19.7% were retired due to disability and 15.7% were not in the labour force. Part/full time employment decreased from 61.4 to 57.1% of the sample 2.5 years later, and 25.5% experienced some change in employment status. Lower level of education and higher level of disability at baseline predicted loss of employment at follow-up. 62.0% of the sample indicated that MS impacted on employment over their lifetime, associated with a lower level of education and progressive MS at time of diagnosis. Retiring due to disability was predictive of a decreased mental health related QOL score. *Conclusion* Employment status was negatively impacted by MS for most participants. We showed for the first time that employment loss was prospectively associated with poorer mental health related quality of life. Employment support including vocational services, reasonable flexibility in the workplace, and legal protection against discrimination should be widely available to assist people with MS, especially for those with progressive onset MS, higher disability and lower levels of education who are at higher risk of employment loss.

## Introduction

Multiple sclerosis (MS) is a chronic neurological condition, often diagnosed in a critical stage of life when people are building their careers. Employment rates are affected almost a decade before formal diagnosis of MS and keep declining thereafter [1] with on average 44% but up to 75% of people with MS (PwMS) reported to be unemployed or retired early [2–5].

The impact of MS on employment is attributable to a range of symptoms including physical disability, problems with cognitive function, fatigue and pain [6–9]. These symptoms may affect employment status in different ways. 90% of PwMS who cut back on hours reported that fatigue was the main cause, while physical disability and cognitive symptoms were chiefly responsible for 86% of PwMS retiring completely due to MS [10]. Discrimination in the workplace, which may lead to either unlawful discharge or failure to provide reasonable accommodations, is also a barrier to adequate employment in PwMS [11, 12]. A 2018 meta-analysis highlighted that while many cross-sectional studies have investigated associations between employment and clinical and demographic characteristics, only two prospective studies have been published in this area, and more studies are needed [13].

Adequate employment has many benefits for mental health, and material wellbeing, even in countries with good social security measures [14]. In contrast, job loss and unemployment is detrimental for mental wellbeing [15, 16], although this relationship is complex and analyses are prone to confounding by socioeconomic factors and reverse causation (whereby poor health causes unemployment) [14, 17]. Unemployment may lead to loss of health insurance and economic stability, and even poverty, which may limit financial capacity for healthy lifestyle, and restrict healthcare choices associated with better health outcomes [18]. Loss of employment further adds to the economic hardship a chronic illness can bring, which may lead to poor health outcomes [18]. Most countries have specialised employment services to support people with disability and chronic conditions to find and retain work and so it is important to understand which populations are most at risk of employment loss to target these services to those who need it most [19]. A recent meta-analyses of 33 cross-sectional studies reported a clear association between better mental health and QOL and full or part time employment in PwMS, however, few prospective studies currently exist [2].

This large international study used two waves of data to prospectively assess changes in employment, determinants associated with this change, and, for the first time, how mental health related quality of life was affected while adjusting for potential confounders. In addition, we assessed which factors at diagnosis were associated with negative impacts to employment at the time of data collection.

## Methods

The St Vincent’s Hospital Melbourne Human Research Ethics Committee (LRR055/12) and the Health Sciences Human Ethics Sub-Committee at the University of Melbourne provided ethical approval for the study (Ethics ID: 1545102).

### Recruitment

English-speaking PwMS, recruited through online MS forums, social media, and MS society newsletters completed the online Health Outcomes and Lifestyle In a Sample of people with MS (HOLISM) study survey in 2012 [20]. After reading the participant information and indicating consent for participation in the study, participants entered the survey which took approximately 40 min to complete. Surveys included items regarding socio-demographic factors, MS characteristics, health outcomes, and lifestyle variables. Baseline participants were contacted by email 2.5 years after initial participation to complete a follow up questionnaire.

### Survey Items

#### Demographic Factors

Demographic variables were collected including age, sex (male, female), marital status (single, partnered), having children (yes, no), and educational status (primary/secondary school, vocational training, bachelor degree, postgraduate degree).

#### Employment

Participants were asked to indicate their current employment status at both time points with multiple choice options which were later collapsed into four categories: (1) employed full time; (2) employed part time; (3) not in the work force or unemployed (included stay at home parent/carer, full-time student, unemployed seeking employment, unemployed not seeking employment, retired due to age); and (4) retired due to disability (retired due to medical reasons/disability). Free text comments were categorised within existing multiple choice categories for the 64 participants who chose to provide information. The employment status variable was used to determine if participants had lost employment, by identifying participants who went from full time at baseline to part-time employment at follow-up; or from full-time or part-time employment at baseline to one of the following statuses at follow-up: stay at home parent/carer, full-time student, unemployed seeking employment, unemployed not seeking employment, retired due to age, or retired due to medical reasons/disability.

The multiple-choice item “how has MS impacted on your employment?” was a single question at 2.5 year follow-up only, covering lifetime (not baseline to follow-up) with 9 multiple choice options which were collapsed into four categories for reporting: (1) no impact (“MS has not impacted on my employment”); (2) had to cut back hours or take increased sick leave (“I have to take increased sick leave due to MS”; “I have had to cut back my hours by approximately 25% due to MS”; “I have had to cut back my hours by approximately 50% due to MS”; “I have had to cut back my hours by approximately 75% due to MS”); (3) changed jobs/tasks or not progressed in (“I have not progressed in my career due to MS”; “I have had to quit or change jobs due to MS”; “I have had to change my day to day tasks at work due to MS”); and 4) had to retire (“I have had to retire completely due to MS”). This outcome variable was further collapsed into a binary variable (impact/no impact on employment) for the purpose of regression analysis.

#### Mental Health QOL Score

The Multiple Sclerosis Quality of Life-54 (MSQOL-54) [21] is widely used to assess health related QOL in PwMS. The MSQOL-54 mental health composite score and the subscale scores for pain and cognitive function (which do not form part of the mental health related QOL composite) were included in analyses as covariates.

#### Clinical Characteristics

Clinical variables were assessed at both time points, with MS related questions including the onset type, type of MS currently diagnosed and disease duration (years since diagnosis). Further, a score of 3 or more on the Patient Health Questionnaire depression module short version (PHQ-2) indicated a positive screen for depression [22]. The fatigue severity scale (FSS) [23], with a cut-off of 4 or more, was used as an indicator of clinically significant fatigue. Level of gait disability was assessed using the Patient Determined Disease Steps (PDDS), a validated self-report version of the commonly used clinician-administered Expanded Disability Status Scale [24], which has ordinal scores from 0 (no disability) to 8 (bed-bound). This was categorised as 0 “no disability” 1–3 “mild disability” and 4–8 “moderate/severe disability”. A list of 24 disease-modifying drugs (DMDs) was provided and participants were categorised into 4 groups: not taking DMD, taking DMD less than 12 months, taking DMD 1–10 years, taking DMD 10 years+.

### Data Analysis

The sample for analysis included all participants who responded to the baseline and follow up surveys. Participants who indicated they did not have their MS diagnosis confirmed by a medical doctor were excluded from all analyses.

**Fig. 1:**
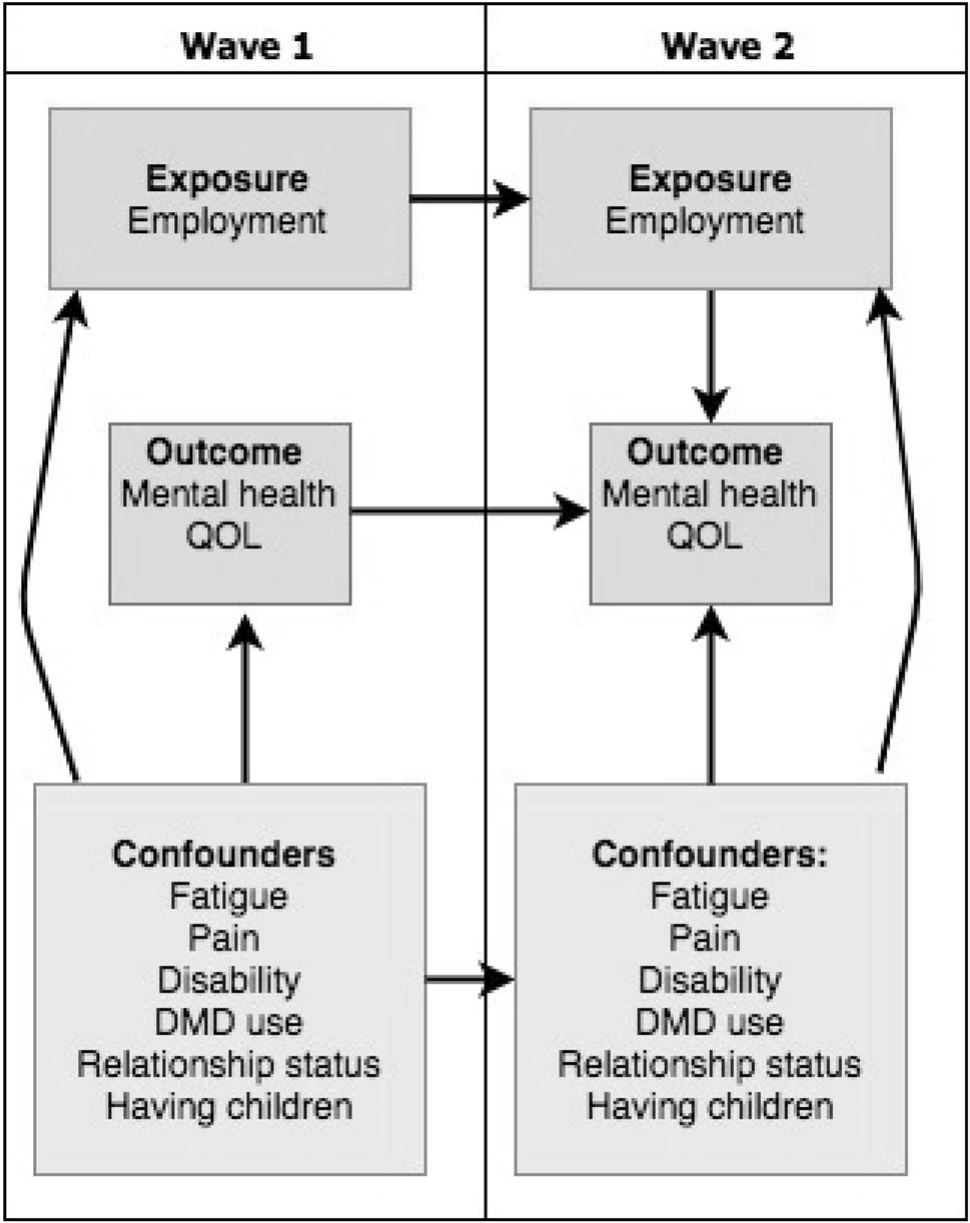
Directed acyclic graph of the association between employment and quality of life (research question 4)

**Fig. 2:**
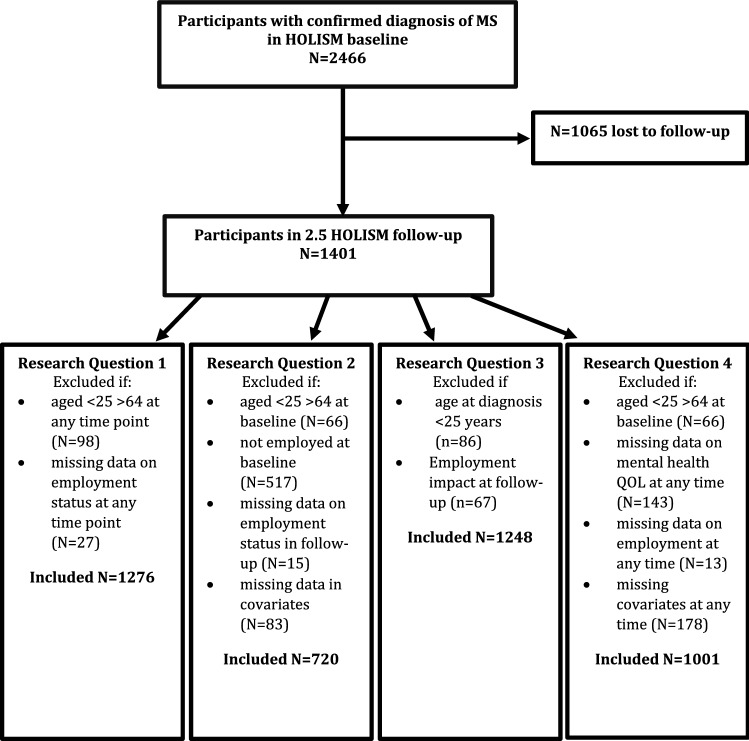
Flow diagram

- Research question 1: *What proportion of people with MS experience a change in employment status after 2.5* *years?* Employment status at baseline and follow up was cross-tabulated to form an employment change matrix.
- Research question 2: *Which baseline factors predict loss of employment at follow*-*up (in people employed at baseline)?* Log binomial regression models were used to assess the association between baselines MS characteristics (cognitive function, depression, fatigue, pain and level of disability) and loss of employment at follow up. Models were adjusted for confounders determined a priori, which included the other disease characteristics, age, sex, education and DMD use at baseline.
- Research question 3: *Which factors at time of diagnosis predict whether MS has a negative impact on employment?* Unadjusted log binomial regression models were run separately for the exposures determined a priori (including age and type of MS at onset, level of education and sex). We did not adjust for confounders in this analysis because we could not assess whether these factors preceded time of diagnosis. Sensitivity analyses were conducted to ensure that the results were not explained by disease duration. While disease duration was associated with age, it was not associated with level of education, sex or type of MS.
- Research question 4: *How is loss of employment associated with change in mental health related QOL?* Fixed-effects linear regression models were used to model the effects of changes in employment on changes in the mental component summary score of the MSQOL. Fixed-effects estimators model the within-individual variation in outcome associated with changes in exposure and therefore implicitly control for time-invariant characteristics [25]. We controlled for potential time-varying confounders determined a priori using a directed acyclic graph (Fig. 1). For inclusion and exclusion criteria for each of the research questions, see Fig. 2.

## Results

Of the 2466 PwMS at baseline, 56.8% completed the follow-up survey (see Fig. 2). Respondents, compared to those lost to follow-up, had higher mental and physical health quality of life, lower levels of disability, shorter disease duration, and were less likely retired due to medical reasons or disability [20].

Participants were mostly female, with a university degree and on average around 45 years of age. Most had relapsing–remitting MS and most reported no or mild mobility disability. Participants were predominantly based in the United States, United Kingdom, Canada, Australia and New Zealand. Table 1 summarises baseline characteristics for the samples within this study for each of the four research questions.

**Table 1:** Baseline characteristics per research question

	Research question 1	Research question 2	Research question 3	Research question 4
	N = 1276	N = 720	N = 1248	N = 1001
Age				
Mean	45.1	43.8	46.5	45.5
SD	9.1	8.9	10.0	9.4
Age at diagnosis				
Mean	37.6	37.3	39.3	37.9
SD	9.2	8.8	9.3	9.5
Sex				
Male				
n	207	149	224	175
%	16.3	20.7	18.0	17.5
Female				
n	1063	571	1024	826
%	83.7	79.3	82.1	82.5
Educational background				
Up to secondary school				
n	253	112	246	194
%	19.9	15.6	19.8	19.5
Vocational training				
n	182	90	183	133
%	14.3	12.5	14.8	13.4
Bachelor degree				
n	497	295	481	408
%	39.2	41.0	38.8	41.0
Postgraduate degree				
n	337	223	330	261
%	26.6	31.0	26.6	26.2
Relationship status				
In a relationship				
n	970	549	945	790
%	76.9	77.2	76.7	78.9
Not in relationship				
n	291	162	287	211
%	23.1	22.8	23.3	21.1
Children				
No				
n	437	277	403	346
%	34.8	39.2	32.8	34.6
Yes				
n	818	430	826	655
%	65.2	60.8	67.2	65.4
Type of MS				
Other				
n	219	125	223	181
%	17.4	17.4	18.1	18.1
Relapsing–remitting				
n	819	517	771	642
%	65.1	72.1	62.5	64.3
Progressive				
n	221	75	239	175
%	17.6	10.5	19.4	17.5
Level of disability				
None/mild				
n	729	511	707	600
%	59.5	71.0	58.6	59.9
Moderate				
n	400	193	395	325
%	32.7	26.8	32.8	32.5
Severe				
n	96	16	104	76
%	7.8	2.2	8.6	7.6

### Change in Employment Status

Of those aged between 25 and 64 years at both time points with available employment data, 35.9% were employed full time, 26.4% were employed part-time, 18.0% were not in the work force, and 19.7% were retired due to disability at baseline (Table 2). Though the proportion employed (full-time and part-time combined) changed only from 61.4 at baseline to 57.1% at follow-up, 325 participants (25.5%) experienced a change in employment status. This includes 169 (13.2%) who lost full or part-time employment, and 94 (7.4%) who gained employment.

**Table 2:** Employment change matrix

Baseline	2.5 year follow up				
	Employed full time	Employed part time	Not in the work force	Retired due to disability	Total^b^
Employed full time	367 (80.1%)	53 (11.6%)	21 (4.6%)	17 (3.7%)	458 (35.9%)
Employed part time	31 (9.2%)	228 (67.7%)	37 (11.0%)	41 (12.2%)	337 (26.4%)
Not in the work force	15 (6.5%)	33 (14.4%)	143 (62.2%)	39 (17.0%)	230 (18.0%)
Retired due to disability	2 (0.8%)	13 (5.2%)	23 (9.2%)	213 (84.9%)	251 (19.7%)
Total^a^	415 (32.5%)	327 (25.6%)	224 (17.6)	310 (24.3)	1276 (100%)

^a^Includes participants aged between 25 and 64 years at both time points with completed employment data

^b^Column percentage is reported here, while row percentage is included in the rest of the table

### Baseline Factors Predicting Loss of Employment

Of those 720 with employment at baseline (full-time and part-time) and complete data on all variables included in the analysis, 151 (21.0%) lost employment and 26 (3.6%) gained employment (part-time to full-time). For those employed at baseline, those with lower education and with higher level of disability at baseline were more likely to lose employment. Older age and being clinically fatigued showed some evidence of predicting loss of employment at follow-up (Table 3).

**Table 3:** Baseline predictors of employment loss at follow-up

	Prevalence ratio	95% confidence interval
Age^a^		
25–34	Ref	
35–44	0.72	0.47, 1.11
45–54	1.08	0.72, 1.62
55–64	1.49	0.96, 2.33
Sex^a^		
Female	Ref	
Male	0.80	0.54, 1.17
Education^a^		
Postgraduate degree	Ref	
Bachelors degree	1.58	1.01, 2.48
Vocational training	1.75	1.10, 2.78
Up to secondary school	1.47	1.01, 2.14
Cognitive function score^b^ (per 10/100 points)	1.00	0.93, 1.07
Positive depression screen^b^	1.06	0.67, 1.69
Clinically fatigued^b^	1.37	0.95, 1.98
Level of disability^b^		
None/mild	Ref	
Moderate	1.55	1.07, 2.26
Severe	2.31	1.44, 3.72
Pain score^b^ (per 10/100 points)	1.00	0.93, 1.07

^a^Unadjusted prevalence ratio

^b^Regression models are adjusted for age, sex, education and disease modifying drug use

### Impact of MS on Employment

A total of 62.0% of the sample (N = 774) indicated that MS has had an impact on their employment; 28.4% of the sample retired due to disability, 17.2% cut back hours or took increased sick leave, and 16.4% changed jobs or reported not progressing in their careers. For each year since diagnosis participants were more likely to report being retired due to MS (prevalence ratio (PR) 1.03; 95% CI 1.02, 1.03), and having changed jobs/tasks or not progressed in career (PR 1.02; 95% CI 1.01, 1.03), but no strong evidence was found for an association between disease duration and having to cut back hours or take increased sick leave (PR 1.01; 95% CI 0.99, 1.03).

### Factors at Diagnosis Predicting Whether MS has a Negative Impact on Employment

Compared to postgraduate education, having vocational training was predictive of employment being impacted by MS (Table 4), but no association was found for sex. Progressive onset MS was only predictive of retiring due to MS while older age at diagnosis was predictive of retiring due to MS, younger age at diagnosis was predictive of cutting hours or taking increased sick leave. When looking at impact of MS as a whole (either retiring, cutting back hours/increasing sick leave or changing jobs/tasks), lower levels of education and progressive type of MS at time of diagnosis were predictive of employment being impacted, but not sex or age at time of diagnosis. Disease duration was not associated with impact on employment, level of education or type of MS and this variable has therefore unlikely confounded these associations.

**Table 4:** Factors predicting impact of MS on employment over lifetime

		Retired due to MS		Cut hours or increased leave		Changed job/tasks or stalled career		MS impacted on employment	
		PR	95% CI	PR	95% CI	PR	95% CI	PR	95% CI
Level of education	Postgraduate	Ref		Ref		Ref		Ref	
	Bachelor	1.19	0.93, 1.53	1.15	0.87, 1.52	1.15	0.87, 1.54	1.10	0.97, 1.25
	Vocational training	2.12	1.68, 2.70	1.51	1.08, 2.13	1.63	1.16, 2.28	1.42	1.26, 1.61
	Up to secondary school	1.19	0.93, 1.52	1.21	0.86, 1.70	1.13	0.78, 1.63	1.29	1.13, 1.47
Sex	Female	Ref		Ref		Ref		Ref	
	Male	0.97	079, 1.19	0.73	0.52, 1.03	0.94	0.70, 1.27	0.94	0.83, 1.06
Type of MS at onset	Relapsing remitting	Ref		Ref		Ref		Ref	
	Progressive	1.68	1.41, 2.00	0.96	0.60, 1.53	1.02	0.65, 1.60	1.22	1.08, 1.37
	Unsure/other	1.06	0.79, 1.44	0.87	0.55, 1.38	0.51	0.26, 1.02	0.92	0.76, 1.12
Age at diagnosis	Under 30	Ref		Ref		Ref		Ref	
	30–39	1.34	0.99, 1.79	0.72	0.55, 0.95	1.24	0.88, 1.74	1.03	0.91, 1.18
	40–49	1.64	1.23, 2.21	0.81	0.60, 1.09	1.30	0.91, 1.85	1.12	0.98, 1.28
	50 and over	1.71	1.26, 2.33	0.67	0.46, 1.00	0.71	0.41, 1.20	1.05	0.90, 1.24

All separate and unadjusted models, comparison group is those who report no impact of MS on employment

*PR* prevalence ratio

**Table 5:** Fixed effects analysis for employment loss and QOL outcomes

	Mental health QOL^a^	
	Adj. β Coef.	95% CI
Employed full time	Ref	
Employed part time	− 2.42	− 5.67, 0.83
Not in the work force	− 2.62	− 6.28, 1.05
Retired due to disability	− 5.42	− 9.54, − 1.29

^a^Adjusted for time-varying confounders disability, fatigue, DMD use, relationship status, number of children, QOL pain score

### Association Between Loss of Employment and Change in Mental Health Related QOL

For those aged 25–64 years with complete data at baseline and follow-up for all variables included in the analysis, we assessed the effects or loss of employment on mental health related QOL. Analyses showed that retiring due to disability compared to being in full time employment was associated with a decline in mental health QOL of more than 5 points (Table 5).

## Discussion

This prospective study found that level of disability and education was predictive of loss of employment 2.5 years later. We did not find evidence that self-reported cognitive function, depression or pain were associated with employment loss over this period although there was weak evidence to suggest that fatigue may play a role. Most participants reported that MS had negatively impacted their career, either through loss of employment, increased sick leave or lack of career progression, despite the majority of participants reporting no or mild mobility disability. Retirement due to medical or disability reasons was associated with a significant decrease in mental health related QOL, adjusting for other clinical characteristics.

The majority of PwMS in our study were employed; 61.4% at baseline and 57.1% at follow-up, with approximately a quarter retired due to disability. Similarly, Fantoni-Quinton et al. report that 68% of their 941 online survey respondents were employed and 27% had retired due to MS [3]. More people lost rather than gained employment, with 4.3% fewer people employed at follow-up 2.5 years later. This is comparable to the 5.4% over 4 years in a similar Australian study [7]. A study from a North American MS cohort (NARCOMS), which included people only slightly older but with on average much longer disease duration than ours, reported that 40% of the sample was employed, and 6% lost employment over a period of 18 months [26].

Our results showed that both lower level of education and progressive type of MS at time of diagnosis were predictive of impact on future employment (over course of illness), but no associations were found with sex or age at diagnosis. Similarly, level of education and level of disability at study baseline (on average 6.5 years since diagnosis) predicted loss of employment 2.5 years later. We did not find evidence that sex, self-reported cognitive function, depression or pain were associated with employment loss 2.5 years later, and the evidence for fatigue and age was weak. This is in line with a recent meta-analysis including 25 (mostly cross-sectional) studies reporting a key role of relapsing–remitting disease course and higher education and, to a lesser extent, fatigue, pain, disease duration and age, on employment status [13].

Honarmand et al. found that unemployment in PwMS was associated with depression severity [27]. We did not find an association between depression and employment loss, but a difference in how this was operationalized may explain this discrepancy. Employment loss in their study was operationalized as unemployment, whereas we also included a change from full to part time work as employment loss. Interestingly, a study by Chiu et al. found that part-time employment was associated with lower levels of depression compared to full time and unemployment [28]. PwMS may choose to avoid stress, which is related to disease activity, by reducing workload or pressure [29]. This illustrates that associations between mental health and employment is a complex interplay and may include factors such as stress in trying to maintain work that may be beyond one’s capacity versus acceptance of alternatives.

Finally, to our knowledge, this is the first study to show prospectively that retiring due to disability was predictive of a decrease in mental health related QOL. It is common for PwMS to quit or lose their job at a specific disease “milestone” either after diagnosis, a relapse, or when entering the progressive phase of MS [30]. Therefore, it can be difficult to disentangle the effects of employment loss on QOL, when there are likely confounding factors. By examining within-person effects, fixed-effects models implicitly control for confounding by time-invariant characteristics, and we were able to control for a range of time-varying confounders, such as changes in MS characteristics. Therefore, our results indicate that employment loss impacts mental health related quality of life over and above the impact of clinical and other characteristics. People with MS who lose employment may need additional mental health assessment and supports at this time, as part of comprehensive care.

Type of employment, not captured in our data, is often related to level of education, and may influence how much flexibility there is in making workplace adjustments to accommodate symptoms. Factors in the work environment that may enable PwMS to stay in their job include flexible or reduced hours or workload when necessary [31], flexible deadlines, being able to take additional breaks or work from home [11]. Social and structural features to support PwMS to find or maintain adequate employment may include good access to health care, welfare provisions, antidiscrimination legislative protection, and vocational services [32]. Therefore, employment loss is likely caused by an interplay of MS-related factors as well as working environment and employer flexibility [30].

In addition to the detrimental effects of unwanted employment loss and early retirement to the mental and physical QOL and financial circumstances of PwMS and their families, there is a large economic disadvantage. Loss of productivity is now responsible for 32% of the costs of MS in Australia ($1.75 billion annually in 2017) [33]. Fortunately, this has reduced from 49% in 2010 [34], likely due to employers providing more work role and/or environment adjustments [35] and improvements in disease modifying therapies [36], enabling people to stay in employment longer. Assisting people to maintain employment will therefore benefit society, PwMS and their families. Playford suggests that employment status in MS may be regarded as a proxy for comprehensive MS management [37], and others have suggested employment as a rehabilitation or health promotion strategy [28]. While small improvements of employment status in PwMS have been reported globally, interventions specifically aimed at assisting PwMS to maintain employment are still not widely available [38]. As part of holistic MS care, people with MS should be offered assistance with understanding their rights, what employment services are available to them, and what reasonable workplace adjustments they can make or request.

### Strengths and Limitations

This study included English-speaking volunteer participants, and those able to use a device on which to complete the online survey. People with severe vision, cognitive or dexterity impairment, or non-English speaking were unlikely to be included in this sample. Further, attrition was significant at 43.2% between baseline and 2.5 year follow-up, which was not at random, likely resulting in attrition bias [20]. As previously described, those who were lost to follow-up were at baseline more likely to have reported more severe disability, lower QOL, and were less likely to be employed [20]. These limitations may result in biased estimates and some associations may be less representative of the entire MS population. Further, self-report of information may not always be reliable, resulting in measurement error. There are inherent issues with assessing cognitive function with a self-reported questionnaire. Specifically in relation to health outcomes associated with employment loss, there is a possibility that unobserved confounders play a role [14]. We had a small proportion of males in the survey, hence we may not have had enough power to detect the impact of sex on employment loss.

It is likely that PwMS in employment are absent due to illness more often than those without MS [9], but absenteeism or presenteeism was not measured. The time frame of the study, 2.5 years, was relatively short to capture change and this resulted in a small sample size with change in employment status. We did not address the small sample (7.4%) in our study gaining employment, but suggest that future studies assess factors enabling PwMS to re-enter the workforce. While we captured retirement due to disability and retirement due to age, there may have been other reasons for retirement (by choice, lifestyle reasons, performance failure), or for working part-time (MS symptoms, carer responsibilities, or volunteering work) that were not captured in our study. Further, the effects may be different per country or job type, however, we were not able to investigate these differences.

One of the strengths of this study is that employment was not operationalized as a dichotomous variable as often done in similar studies which include students and carers as unemployed [13]. Rather we have categorised participants as full time, part time, unemployment or not in the work force. Further, the prospective nature of this study, and ability to adjust for a range of confounders further strengthens this study, and for the first time assess impact of job loss on mental health related QOL.

## Conclusion

Most participants reported that MS had impacted their employment. People without tertiary education, progressive onset MS or more severe disability were more likely to experience a negative impact. Those with a university degree were less likely to experience negative impact, perhaps because they have less labour intensive jobs, or more flexible work arrangements. This prospective study is the first to show that retiring due to disability was associated with a decline in mental health related QOL. Literature shows that employment support, including vocational services, reasonable flexibility in the workplace, and legal protection against discrimination, should be widely available to assist people with MS. Our results show that these are especially needed for those at higher risk, to prevent loss of employment where possible.
